# Brain-wide atlas of outputs and inputs of mouse auditory cortex revealed by modified viral labeling strategies

**DOI:** 10.1016/j.fmre.2025.08.014

**Published:** 2025-09-08

**Authors:** Junxia Pan, Chaoyue Yang, Ruijie Li, Jialin Li, Pei Sun, Yuanyang Liu, Ke Liu, Xiang Liao, Hongbo Jia, Zhengping Yu, Xiaowei Chen, Meng Wang

**Affiliations:** a Brain Research Center and State Key Laboratory of Trauma and Chemical Poisoning, Third Military Medical University, Chongqing 400038, China; b Department of Occupational Health, Third Military Medical University, Chongqing 400038, China; c Center for Neurointelligence, School of Medicine, Chongqing University, Chongqing 400030, China; d Leibniz Institute for Neurobiology (LIN), Magdeburg 39118, Germany; e Suzhou Institute of Biomedical Engineering and Technology, Chinese Academy of Sciences, Suzhou 215163, China; f Institute of Neuroscience and the SyNergy Cluster, Technical University of Munich, Munich 80802, Germany; g Center for Behavioral and Brain Science (CBBS), Otto von Guericke University, Magdeburg 39120, Germany; h Chongqing Institute for Brain and Intelligence, Guangyang Bay Laboratory, Chongqing 400064, China; i Key Laboratory of Major Brain Disease and Aging Research (Ministry of Education), Chongqing Medical University, Chongqing 400016, China; j Institute for Brain Science and Disease, Chongqing Medical University, Chongqing 40016, China

**Keywords:** Brain networks, Soma labeling, Auditory cortex, Corpus callosum, Connectivity

## Abstract

Understanding brain-wide connectivity is necessary to understand the principles of neural functions. While traditional neuron labeling methods have contributed to structural mapping, they often fall short in accurately identifying neuronal outputs because of limitations in solely using axon tracing. In this study, we improved labeling specificity by incorporating nuclear-localized fluorescent markers into retrograde and monosynaptic anterograde viral tracing strategies. Combined with high-resolution whole-brain imaging, this method enabled precise mapping of input-output relationships in the mouse auditory cortex (ACx). Soma-based labeling allowed for the clear identification of upstream and downstream neurons across the entire brain. Our analysis revealed that ACx projections to the primary somatosensory (SSp) and primary visual (VISp) cortices were largely restricted to layer 5, contrasting with previous reports suggesting stronger innervation in layers 1–3. We also identified downstream neurons within the corpus callosum (CC), a region not previously known to receive direct input from the ACx. These findings highlight the advantages of soma-targeted approaches in connectivity research and offer new insights into the organization of auditory networks. This approach lays the groundwork for future studies of circuit morphology and function across the brain.

## Graphical abstract

**Figure:**
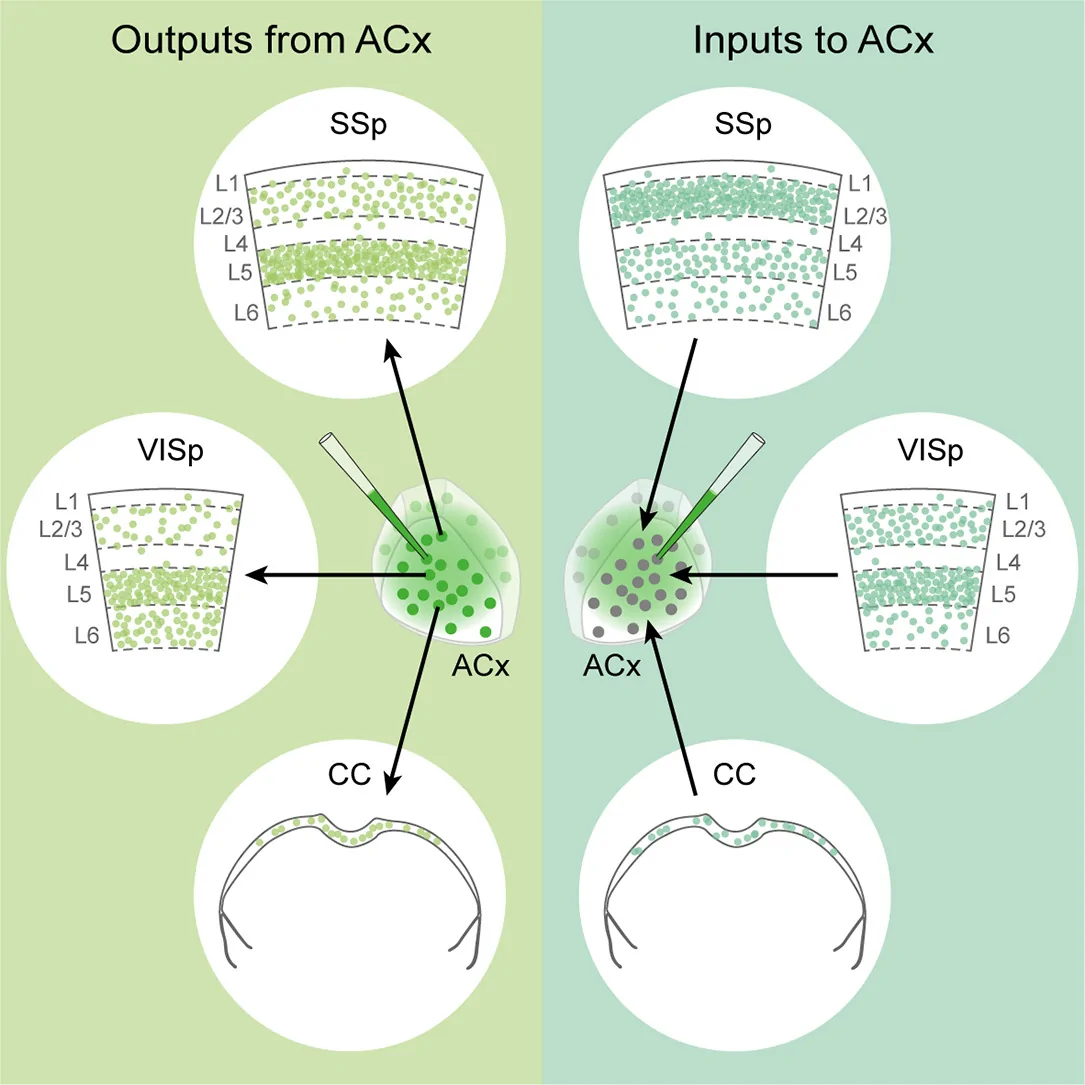

## 1 Introduction

In the mammalian brain, cortical neurons form extensive networks by sending outputs to and receiving inputs from both cortical and subcortical regions [1]. Mapping these networks with high accuracy is needed to understand how neural circuits support brain function. Viral tracers have become a widely used tool for this purpose. For example, the Allen Mouse Brain Connectivity Atlas used adeno-associated viral (AAV) vectors expressing enhanced green fluorescent protein (EGFP) to trace axonal projections from defined brain regions and cell types. Paired with high-throughput serial two-photon tomography, this strategy enabled whole-brain visualization of EGFP-tagged axons [2]. Similarly, Zhao et al. combined anterograde and retrograde viral tracing in the mouse primary auditory cortex, generating comprehensive datasets using a dual-channel fluorescence micro-optical sectioning tomography system [3].

Despite these advances, conventional axon-based tracing methods face inherent limitations. They often cannot differentiate between true axonal terminals and fiber pathways, which can lead to overestimates of connectivity when signal quantification is based solely on the number of EGFP-positive pixels in a region. Retrograde transsynaptic viral tracers, such as rabies virus and pseudorabies virus (PRV), have been widely used to map presynaptic inputs to transduced neurons [[4], [5], [6]]. However, reliable anterograde transsynaptic tracing remains more challenging. While viruses like herpes simplex virus (HSV) [7] and vesicular virus (VSV) [8] have shown potential for anterograde labeling, their neurotoxicity and unpredictable synaptic spread limit their practical use. In 2014, the discovery that the adeno-associated virus of serotype 1 (AAV1) could undergo anterograde transport to axonal terminals [9,10], and subsequent work by Zingg et al. confirmed its transsynaptic capabilities. Compared to AAV1-hSyn-Cre, scAAV1-hSyn-Cre demonstrated superior orthodromically transsynaptic labeling capabilities [11,12]. Concurrently, Tervo et al. developed rAAV2-retro, a retrograde vector engineered for high-efficiency access to projection neurons, compared to synthetic retrograde tracers [13]. Together, these viral tools have significantly improved our ability to map connectivity with both spatial precision and synaptic specificity.

In this study, we used these developments by combining retrograde and monosynaptic anterograde viral tracing with nuclear-localized labeling elements. Using the mouse auditory cortex (ACx) as a model system, we mapped input-output relationships at both regional and laminar resolution. High-resolution fluorescence micro-optical sectioning tomography [14] allowed us to visualize the spatial distribution of labeled somata across the entire brain. This soma-centered approach enabled the accurate quantification of projection patterns and revealed several key findings. For example, layer 2/3 neurons in the primary somatosensory cortex preferentially projected to ACx, while layers 5 and 6b received projections from the ACx [15,16]. Notably, these laminar patterns differed from those reported using conventional axonal labeling approaches. Additionally, we identified previously unreported output and input somata within the corpus callosum, the major nerve tract connecting the two cerebral hemispheres, which is not typically associated with direct ACx connectivity.

Together, these results underscore the value of soma labeling for developing our understanding of long-range neuronal connections. By improving both the spatial accuracy and interpretability of brain-wide connectivity maps, our approach offers a robust tool for understanding cortical circuits and their roles in sensory processing.

## 2 Materials and methods

### 2.1 Animals

All animal experiments in this study were approved by the Animal Care and Use Committee of Third Military Medical University (Approval number: AMUWEC20211009) and performed in compliance with institutional guidelines for animal welfare. Male C57BL/6 J mice (8 weeks old) were obtained from the Laboratory Animal Center at the same institution. The mice were housed under controlled conditions, with a room temperature of 25 °C, ad libitum access to food and water, and a 12-h light/dark cycle (light phase: 7:00 a.m.−7:00 pm.).

### 2.2 Virus and reagents

The scAAV2/1-hSyn-H2B-mclover3-pA and AAV2/2Retro Plus-hSyn-EGFP.Nuc-WPRE-pA were purchased from Taitool Inc. (Shanghai, China). Cholera toxin subunit B, Alexa Fluor 488 conjugate, and propidium iodide were obtained from Invitrogen (C34775; P1304MP). Lowicryl HM20 resin was sourced from Electron Microscopy Sciences (RT-14340). Isoflurane was provided by RWD Life Sciences (Shenzhen, China).

Stereotaxic virus injections were carried out following our previously described procedures [17]. Briefly, mice were anesthetized with 1.0% isoflurane, and craniotomy was performed using a stereotaxic apparatus (#68030, RWD Life Science, China). For anterograde tracing, 150 nL of scAAV2/1-hSyn-H2B-mclover was injected into the ACx of wild-type mice at coordinates bregma −3.0 mm, −4.5 mm lateral to midline, and −2.2 mm depth from the skull surface, according to *The Mouse Brain in Stereotaxic Coordinates* [18]. For retrograde tracing, 150 nL of AAV2/2Retro Plus-hSyn-nuclear-EGFP was injected. Following the injection, the pipette was held in place for 5 min before retraction. The incision was then sutured.

### 2.3 Tissue embedding

Thirty days post-injection, mice were deeply anesthetized with 1.0% pentobarbital sodium and transcranially perfused with 0.01 M phosphate‐buffered saline (PBS; cat. no. P3813–10PAK, Sigma-Aldrich), followed by 4% paraformaldehyde (PFA; Sigma-Aldrich, cat. no. P 6148–1KG) in 0.01 M PBS. Extracted brains were post-fixed in PFA at 4 °C overnight before resin embedding using Lowicryl HM20 (Electron Microscopy Sciences, cat. no. 14340), with minor modifications from previously reported methods [19].

Brains were washed three times in 0.01 M PBS over 24 h, then dehydrated using a graded ethanol series (50%, 75%, 95%, and two changes of 100%), with each step lasting 2 h. Following dehydration, the tissue was infiltrated with increasing concentrations of resin dissolved in ethanol (50%, 75%, and two changes of 100%). Each of the first three steps was carried out for 2 h, while the final infiltration in 100% resin was extended to 48 h. After infiltration, samples were embedded in gelatin capsules filled with fresh resin and polymerized at 50 °C for 8 h.

### 2.4 Imaging

The embedded brain samples were sealed at the center of a bath containing 0.05 M Na_2_CO_3_ (cat# 222321–500 G, Sigma-Aldrich) to reduce background fluorescence and stained with propidium iodide (2mg/ml; cat# P1304MP) for cytoarchitecture visualization [20]. Imaging was performed using a fluorescence micro-optical sectioning tomography (fMOST) system (BioMapping5000, Wuhan OE-Bio Co., Ltd.), with a resolution of 0.3 µm × 0.3 µm × 2 µm. Whole-brain scans were acquired in the coronal plane, capturing GFP and PI channels. The brain was subsequently sectioned into 1-µm slices using a diamond knife [21,22].

### 2.5 Image registration

Whole-brain imaging data were aligned to the Allen Common Coordinate Framework version 3 (CCFv3). PI channel data were downsampled to a spatial resolution of 10 µm × 10 µm × 10 µm. Registration involved several steps: initial rigid alignment, followed by greyscale-based 3D affine registration to the Allen Brain Atlas, and finally, dense landmark-based 2D registration in localized regions. Stable anatomical features, such as the ventricles, hippocampus, and corpus callosum, served as reference landmarks for registration and boundary determination [21].

Regional boundaries were identified by using differences in cell density, morphology, and other features that distinguish cortical and subcortical regions. These features were registered to the atlas using grey-level-based algorithms. The downsampled dataset was then registered into the Allen Reference Atlas, establishing a correspondence between the acquired images and the Allen CCFv3 brain atlas. All registration procedures were executed using Elastix software [23]. The resulting transformed parameters were applied to reconstruct the traced somas within the Allen Brain Atlas framework. The accuracy of the registration was independently validated by two analysts.

### 2.6 Soma detection

The detection system consisted of a classification module designed to identify regions containing somata within volumetric images and a detection module tasked with accurately localizing these somata in 3D space. Once the somata were identified and localized, their coordinates were registered with the Allen Brain Atlas to facilitate brain function mapping and analysis [24].

### 2.7 Visualization and statistical analysis

A custom Python script was used to extract registered somata from designated brain regions. Data are presented as mean ± standard error of the mean (SEM). Statistical analyses were carried out in MATLAB 2012a (MathWorks). Group comparisons were performed using two-sided Mann-Whitney U tests and two-sided Welch’s *t*-tests, as appropriate.

## 3 Results

### 3.1 Whole-brain imaging of virus-assisted tracing of neural output and input circuits

AAV1 exhibits strong anterograde transsynaptic properties, with high-titer viral vectors effectively labeling neurons in downstream brain regions [11,25]. To trace whole-brain connections of the ACx, we marked the somata of afferent and efferent neurons in male C57BL/6 J mice.

For output mapping, a self-complementary AAV1 carrying a nuclear-localized fluorescent protein under the control of the human synapsin promoter (scAAV2/1-hSyn-H2B-mclover3-pA, hereafter referred to as scAAV-mclover) was utilized (Fig. 1a). For input mapping, a retrograde AAV carrying nuclear-localized enhanced GFP under the same promoter (AAV2/2Retro Plus-hSyn-EGFP.Nuc-WPRE-pA, hereafter referred to as AAVRetro-EGFP) (Fig. 1b) [26]. To evaluate the precision of retrograde and anterograde viral tracing, we quantitatively analyzed the spatial distribution and volume of injection sites across samples. Injection site localization was verified using frozen-section histology [27,28]. Representative coronal sections from an example mouse show targeted placement of the retrograde tracer CTB-488 within the auditory cortex (ACx; Fig. 2a), with the majority of starter neurons localized to auditory cortical regions including the primary auditory cortex, the ventral and dorsal auditory cortex (Fig. 2b).

**Fig. 1:**
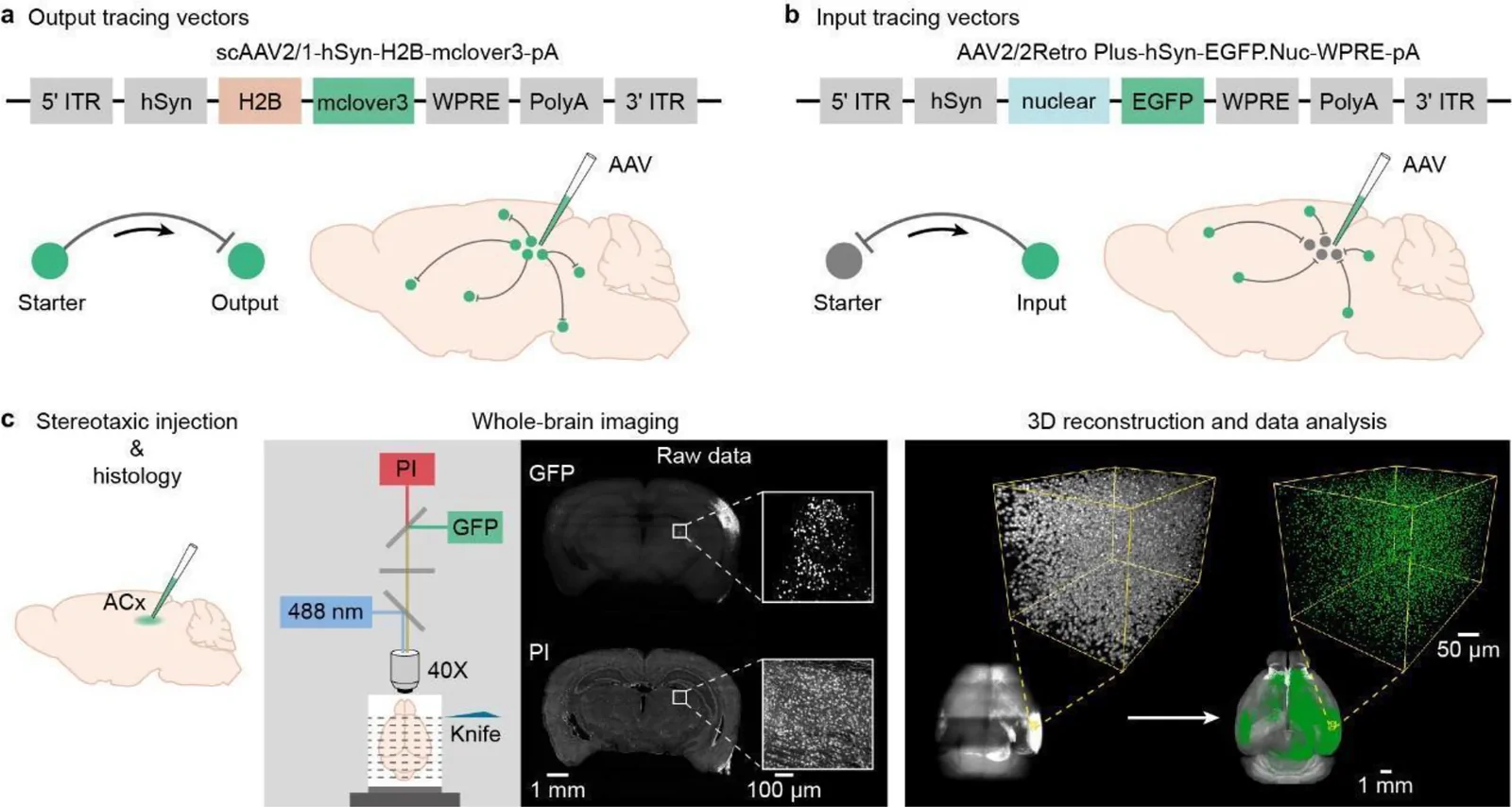
**Schematic of experimental design, imaging, and data analysis pipelines.** (a) Schematic of output tracing vectors (top, scAAV2/1-hSyn-H2B-mclover3-pA) and viral injection procedure (bottom). (b) Schematic of input tracing vectors (top, AAV2/2Retro Plus-hSyn-EGFP.Nuc-WPRE-pA) and viral injection procedure (bottom). (c) The main steps are stereotaxic injection (left), data acquisition (middle), and analysis for anterograde and retrograde tracing (right).

**Fig. 2:**
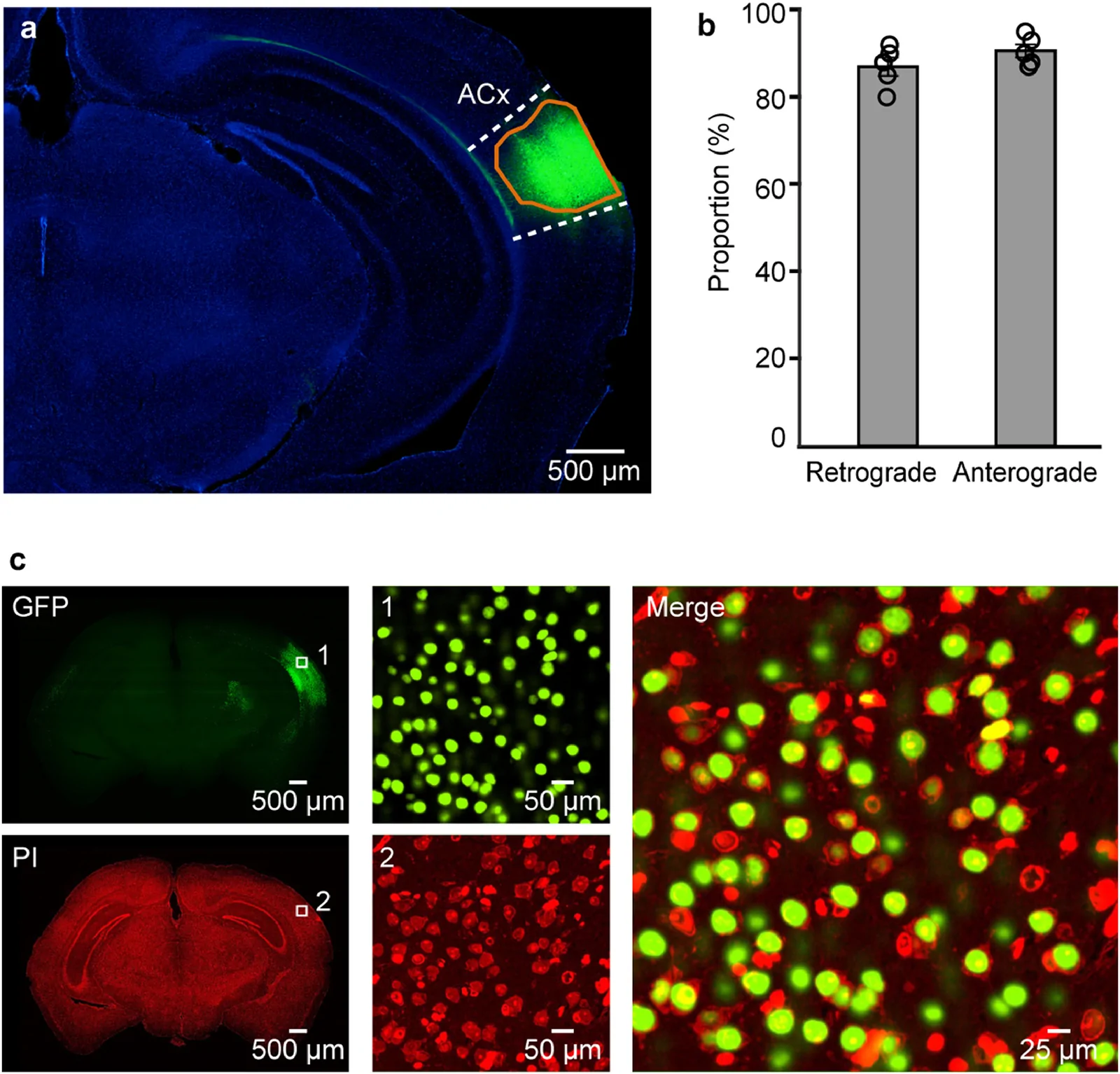
**ACx labeling site and whole-brain dual-color imaging.** (a) A coronal brain slice (40 µm projection) showing the injection site of the retrograde neuronal tracer CTB-488 (in ACx) in an example mouse. (b) Quantitative comparison of starter neuron proportions and infected volumes within ACx between retrograde (*n* = 5) and anterograde (*n* = 5) tracing paradigms. (c) Whole-brain dual-color imaging using fMOST. Left: Maximum intensity projections of two serial coronal sections in the GFP channel (top) and PI channel (bottom). Middle: Enlarged views of the areas outlined by the yellow dashed boxes on the left, with the two channels shown separately. Right: Merged image of the middle panel.

To ensure inter-animal consistency, samples exhibiting excessive variability in either the proportion of starter neurons or infection spread parameters (defined as infected volume exceeding 130% or falling below 70% of baseline values) were designated as contaminated outliers and subsequently excluded from analysis (Fig. 2b).

After 30 days of viral expression, brain samples were imaged using the fMOST system, which enabled continuous, uninterrupted sectioning and eliminated artifacts typically caused by manual sectioning (Fig. 1c). Dual-color imaging was used, with one channel recording GFP-labeled neurons, while the other observed cytoarchitecture using propidium iodide (PI) staining. Whole-brain datasets were acquired from ten samples at a voxel resolution of 0.3 µm × 0.3 µm × 2 µm (Fig. 2c). Imaging of each sample was completed within one week, producing approximately 2 terabytes of raw data per brain. Preprocessed images were reconstructed into coronal sections for downstream 3D analyses (Fig. 1c).

### 3.2 Distribution of brain-wide connectivity outputs of the auditory cortex

Whole-brain cell counting was performed on the imaged brain samples using NeuronMapper, a tool built on a four-stage pipeline: preprocessing, classification, detection, and mapping. In the preprocessing stage, the full imaging dataset was divided into 3D sub-blocks. A lightweight classification model was then applied to identify sub-blocks likely to contain somata. For those identified blocks, soma regions were segmented using a network based on the Video Swin Transformer-based architecture. The resulting soma coordinates were extracted and mapped to the Allen Brain Atlas to enable whole-brain neuron mapping. The positions of labelled neurons were aligned and registered to the Allen Mouse Common Coordinate Framework (CCF v3).

The output signals of the ACx were widely distributed across multiple brain regions, spanning approximately 8.5 mm (ranging from bregma +2.5 mm to bregma −6.0 mm) (Figs. 3 and S1). In alignment with the mesoscale projections of the ACx identified by the Allen institute and previous studies, the densest outputs were observed in the following brain regions: the somatomotor areas (MO), orbital area (ORB), anterior cingulate area (ACA), somatosensory area, including primary (SSp) and supplemental parts (SSs); temporal association areas (TEa), ectorhinal area (ECT), visual areas (VIS); dorsal striatum (i.e. caudoputamen, CP); medial geniculate complex (MG), including medial (MGm), dorsal (MGd), and ventral (MGv) parts; superior colliculus (SC) and inferior colliculus (IC) (Figs. 3b, c and S4, S5).

**Fig. 3:**
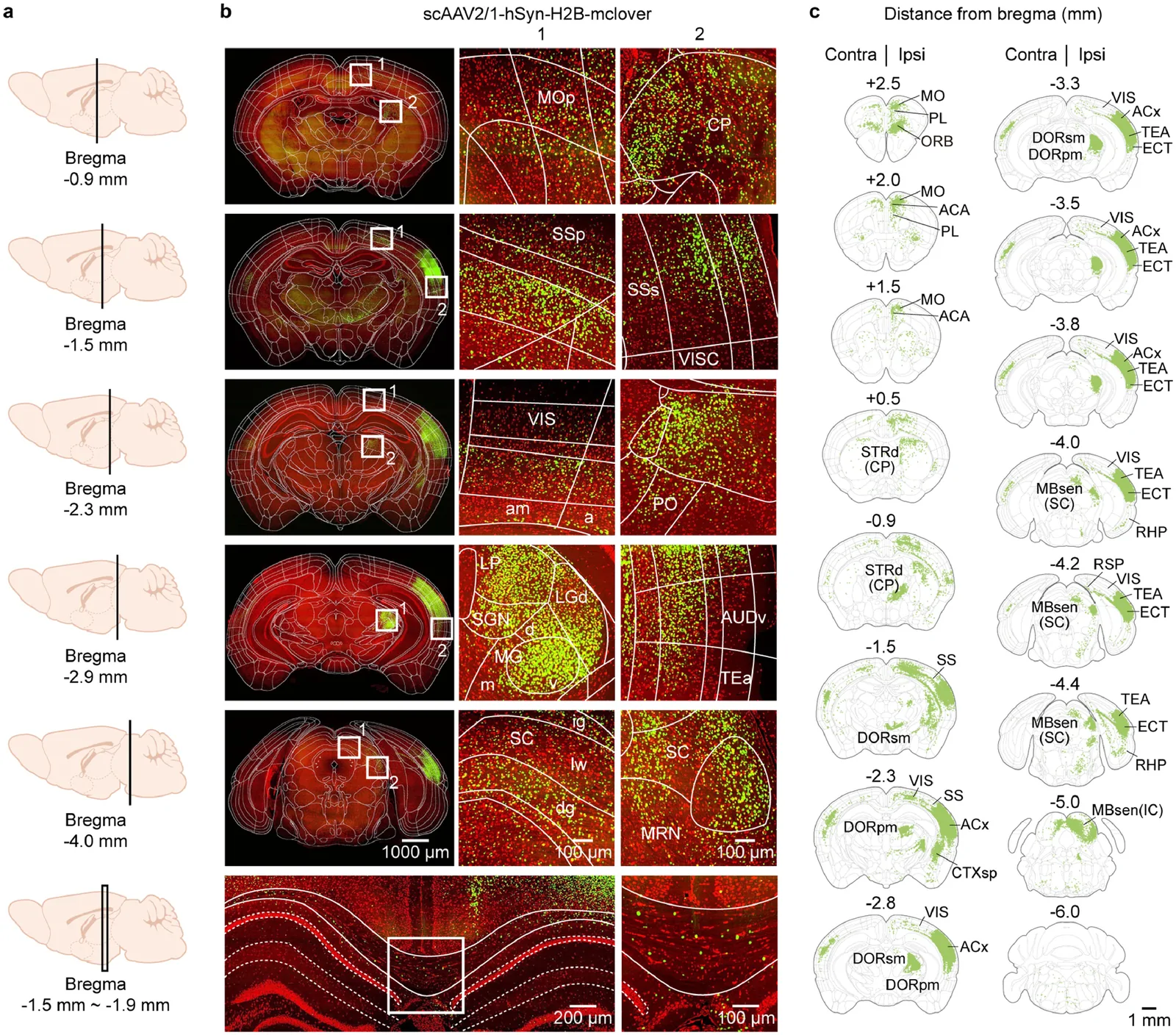
**Representative coronal sections showing output regions from the auditory cortex.** (a) Schematic of sagittal brain outlines indicating approximate distances from the bregma of six representative brain sections. (b) Left: Six dual-color composite coronal sections corresponding to (a) were selected from a representative output whole-brain dataset labeled by scAAV-mClover, showing distributions of postsynaptic cells in the auditory cortex. Maximum intensity projections of green signals were 50 µm in thickness (for lanes 1–5) and 400 µm in thickness (for lane 6). Maximum intensity projections of red signals were 2 µm in thickness for all images. Middle and right: Enlarged views of the respective yellow solid boxes showing signal details. All coronal sections were registered to standard templates of the Allen Brain Atlas. (c) Sixteen coronal sections from a representative output whole-brain dataset were arranged rostrally to caudally with a 50 µm interval (ranging from bregma +2.5 mm to bregma −6.0 mm), illustrating the overall distributions of postsynaptic neurons of the ACx across diverse long-range projecting areas. All coronal brain outlines were adapted and modified from templates of the Allen Brain Atlas. Green dots represent signals of GFP-labeled postsynaptic neurons.

However, monosynaptic soma labeling provided a more accurate, layer-specific depiction of output networks. For instance, in the primary motor area (MOp) and secondary motor area (MOs), scAAV-mclover labeling revealed relatively abundant innervation to MOp in layers 5 and 6, with the densest innervation in layer 5. Similarly, dense innervation was observed in MOs within layers 5 and 6 (Fig. 3b). In contrast, traditional fiber labeling indicated sparse innervation, primarily in layer 1 and less frequently in layers 5 and 6 of MOp. Consistent with previous findings, projections from ACx to SSp were observed across all cortical layers (layers 1–6), with the densest projections in layer 5, while layers 1–3 exhibited only sparse innervation (Figs. 3b, c, 4a). This finding contrasts with earlier reports suggesting that layer 2/3 received the densest projection, followed by moderate levels in layer 5.

**Fig. 4:**
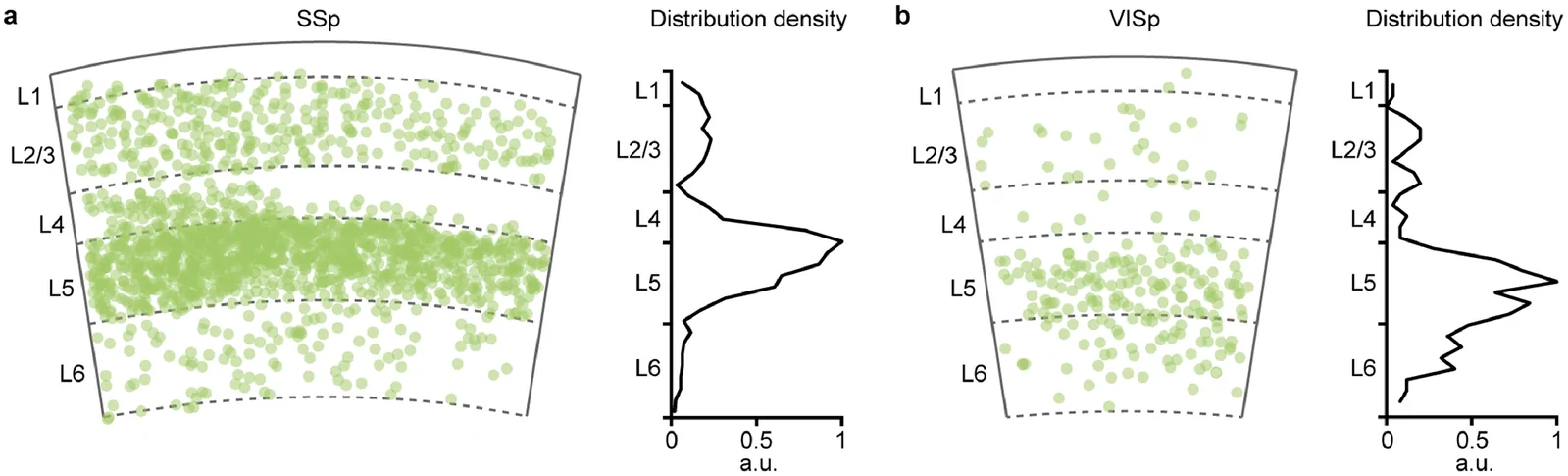
**Laminar distribution of downstream neurons in SSp and VISp.** (a) Left: Ddistribution of labeled somata across cortical layers in the SSp. Right: Qquantification of soma density from the pia to L6 in SSp. (b) Left: Ddistribution of labeled somata across layers in the VISp. Right: Ddensity profile showing the number of downstream somata as a function of depth from the pia to L6 in VISp.

Similar patterns were observed in other cortical regions, such as VISp, where most postsynaptic neurons labeled by scAAV-mclover aggregated in layer 5, with minimal signals in layers 1–3 (Figs. 3b, c, 4b). The Allen Institute’s data showed an opposite pattern, with the densest projections in layers 1–3 and extremely sparse projections in layer 5. Moreover, discrepancies in deeper brain areas were identified. While confirming the abundant outputs to the MGv, our study also identified similarly extensive outputs to the lateral posterior nucleus of the thalamus (LP) as well as localized projections in the SC and IC. The ACx primarily projects to the MGv, with notably higher projection density than other thalamic nuclei (Fig. 3b; Table S2). This projection pattern aligns with the MGv’s role as a hierarchical relay station for auditory information processing.

Interestingly, within the whole-brain dataset labelled by scAAV-mclover, a small fraction of postsynaptic neurons was traced in the corpus callosum (cc), which had previously been reported by the Allen Institute to contain mostly dense contralateral-projecting axons with few neurons. This suggests potential intermediate information transfer during contralateral communication.

Together, these results indicate that the whole-brain imaging approach using a virus-assisted circuit tracing strategy provides a more comprehensive understanding of the brain-wide outputs of the ACx (Fig. 3).

### 3.3 Distribution of brain-wide connectivity inputs to the auditory cortex

To capture a comprehensive view of brain-wide inputs to the ACx, we used an improved Retro-AAV vector encoding nuclear-localized enhanced GFP. This allowed for precise, three-dimensional mapping of upstream neurons. Coronal sections from the whole-brain dataset were processed using a 50 µm maximum intensity projection along the anterior-posterior axis and then aligned to templates from the Allen Brain Atlas. Input neurons were detected in multiple brain regions, including widespread areas of the cerebral cortex, striatum, thalamus, midbrain, and hindbrain. These labeled inputs spanned nearly 8.5 mm, ranging from bregma +2.5 mm to −6.0 mm (Figs. 5 and S2) reflecting the extensive network of the ACx within the brain.

**Fig. 5:**
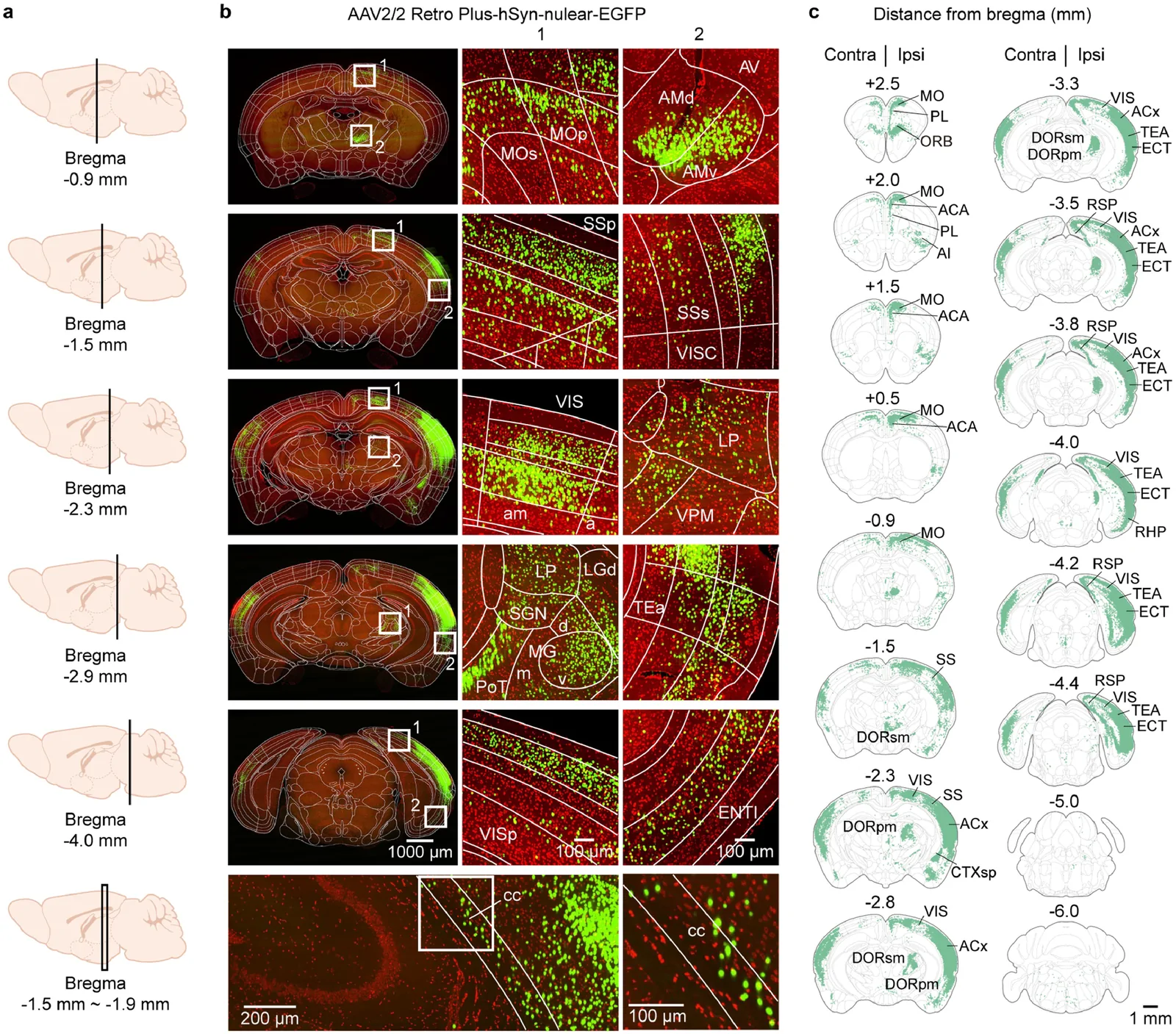
**Representative coronal sections show inputs to the auditory cortex.** (a) Schematic of sagittal brain outlines showing approximate distances from bregma of five representative brain sections. (b) Left: Six dual-color composite coronal sections (green, 50 µm thickness; red, 2 µm thickness) corresponding to (a) were selected from a representative input whole-brain dataset labeled by AAVRetro-EGFP, showing the distribution of presynaptic cells in the auditory cortex. Maximum intensity projections of green signals were 50 µm thick (lanes 1–5) and 400 µm thick (lane 6). Middle and right: Enlarged views of respective yellow solid boxes showing signal details. All coronal sections were registered to standard templates from the Allen Brain Atlas. (c) Sixteen coronal sections from a representative input whole-brain dataset were arranged rostrally to caudally at 50 µm intervals (ranging from bregma +2.5 mm to bregma −6.0 mm), showing the overall distribution of presynaptic neurons of the ACx across diverse long-range projecting areas. All coronal brain outlines were adapted and modified from the Allen Brain Atlas templates. Green dots represent GFP-labeled presynaptic neurons.

In line with mesoscale input mapping of the ACx by the Allen Institute, the temporal association areas (TEa) provided the most significant innervation, apart from intrinsic ACx connections, followed by the SS and VIS areas. In subcortical regions, the lateral striatum dorsal region (STRd) and the medial geniculate complex (MG), including its medial (MGm), dorsal (MGd), and ventral (MGv) subdivisions, exhibited reciprocal connectivity with the ACx. Among these, the MGv was the primary source of thalamic input to the ACx, with dense input clusters in MGv and MGd and sparse input in MGm. These projections showed clear laminar preferences, with tightly clustered labeling in the MGv and MGd, in contrast to the diffuse patterns in the MGm. These findings indicate a dense network of cortical and subcortical connections with the ACx.

Additionally, distinct laminar-specific input patterns to the ACx were observed. In the primary and secondary motor areas (MOp and MOs) at bregma −0.9 mm, layer 5 primarily innervates the ACx. In the SSp at bregma −1.5 mm, layers 2/3 were the main contributors. In contrast, in the VISp at bregma −2.3 mm, layer 5 neurons dominated. The ENTI at bregma −4.0 mm primarily contributed layer 3 neurons. Interestingly, in the somatosensory area, supplemental parts (SSp) at bregma −1.5 mm, inputs originated mainly from layers 2/3 and 5, with minimal contributions from layer 4 (Fig. 5b). Additionally, sparse input somata were identified in the cc (Fig. 5b). These results highlight region-specific contributions to the ACx from diverse cortical areas.

### 3.4 Brain-wide quantification and bias analysis

To comprehensively interpret the brain-wide connectivity of the ACx, we divided the acquired 3D data into 53 gross brain regions according to previously established criteria and quantified upstream and downstream neurons along the axes [29,30]. To account for sample size and imaging variability, we normalized neuron counts by dividing each subregion’s neuron count by the total neuron count per sample. This allowed for the quantification of neurons in each subregion and total input and output neurons across the whole brain (Fig. S3). Whole-brain analysis revealed that regions with the highest input-output correlation were predominantly localized within the cortical-thalamic network. Among subcortical regions interacting with the ACx, the thalamus emerged as the second major hub based on projection density and neuronal input volume (Fig. 6). Notably, the MGv demonstrated strong reciprocal connectivity with ACx neurons (Figs. 3b, 5b). Efferent projections from the ACx were broadly distributed across MGv and MGd, while afferent inputs displayed a more restricted topography, with dense labeling in the dorsal MGv and MGd and minimal input from MGm. These non-reciprocal connectivity patterns between the auditory cortex (ACx) and thalamic nuclei align with previously reported network architectures in sensory processing systems [31].

**Fig. 6:**
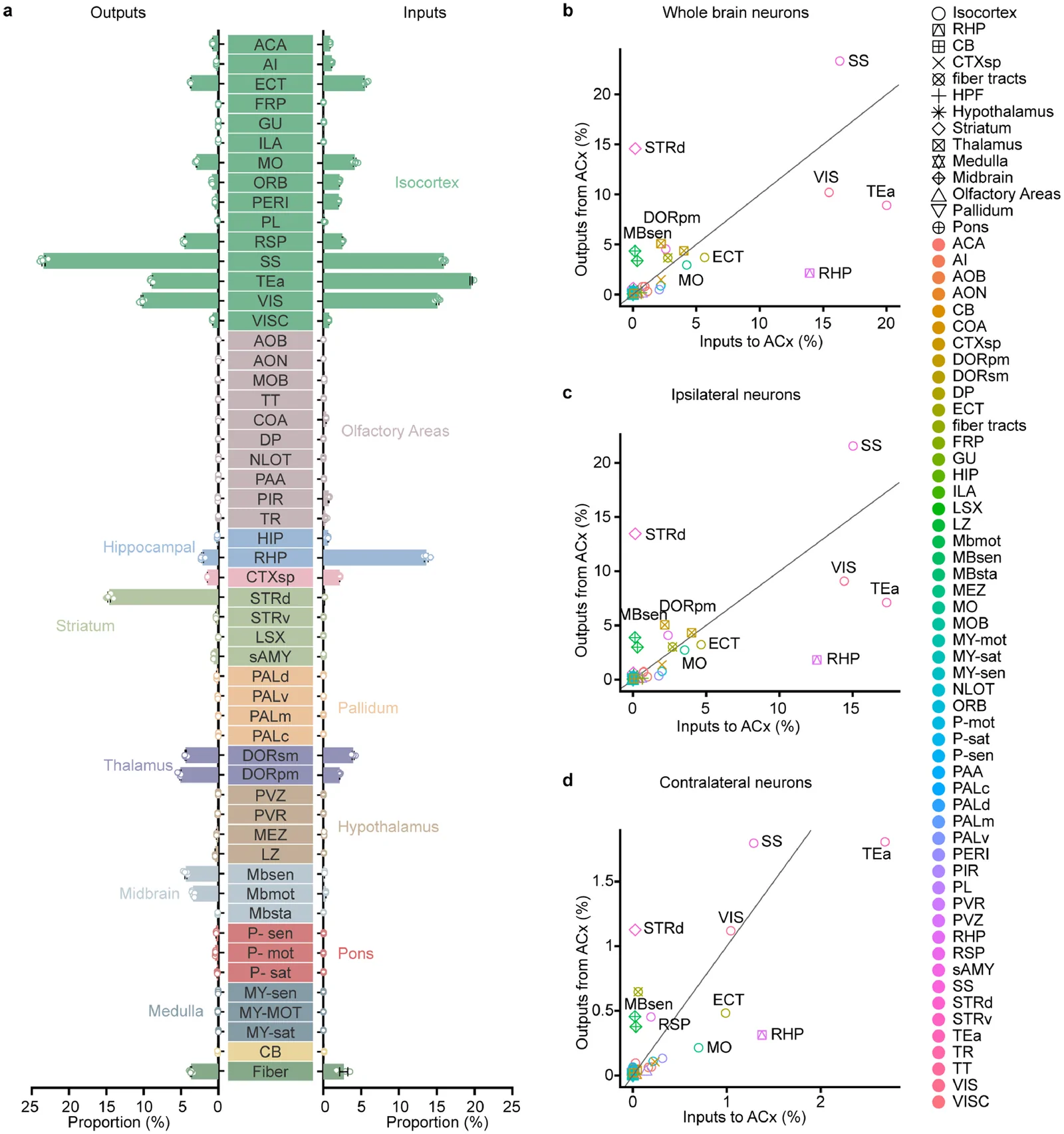
**Quantitative analysis of whole-brain outputs and inputs**. (a) Quantitative characteristics of outputs and inputs across different brain regions. The brains were divided into 13 gross regions for both groups. The percentage of outputs in diverse subregions was calculated as the number of output neurons in a given subregion divided by the total number of output neurons (for the scAAV-mclover labelled group). Similarly, the percentage of input neurons in a given subregion is divided by the total number of input neurons (for the AAVRetro-EGFP labelled group). (b-d) Cooperativity and Bias Analysis: (b) Comparison of brain-wide outputs and inputs of the ACx; (c) Comparison of outputs and inputs of the ACx in the ipsilateral hemisphere; (d) Comparison of outputs and inputs of the ACx in the contralateral hemisphere.

In the isocortex, input and output distributions followed similar patterns, though some regions displayed distinct biases. For instance, output somata were abundant in the STRd and IC, whereas input somata in these regions were sparse (Fig. 6a). Most connections were ipsilateral, with contralateral connections primarily located within the isocortex. Medial–lateral (ML) distribution analysis showed that 90.1% of outputs and 86.8% of inputs were ipsilateral (Fig. S5; Tables S1-S3), consistent with prior findings [3].

Although most regions exhibited both input to and output from the ACx, their distributions varied. For example, inputs from MOp and MOs were mainly found in layer 5, while outputs to these areas are more widely distributed across layers 2 to 6 (bregma −1.5 mm). In the SSp, inputs were concentrated in layers 2/3 and 5, whereas outputs were denser in layer 5 and extended into layer 6 (bregma −1.5 mm). Previous studies have reported that the somatosensory cortex responds to auditory stimuli during tasks involving sound and touch, with auditory input potentially enhancing somatosensory encoding [[32], [33], [34], [35]]. In the VISp, inputs were dense in layers 2–5, whereas outputs predominantly originated from layer 5 (bregma −2.3 mm). Real-world stimuli often contain multiple sensory features, suggesting that specific stimuli may engage neuronal populations to generate synergistic responses. Visual stimuli can modulate auditory cortex neuron firing based on stimulus efficacy and timing [36,37].

To better understand the functional connectivity, we assessed regional input-output strength biases. Several areas, including the SS and retrosplenial (RSP) in the isocortex, as well as regions within the hippocampus (HPF), MB, and thalamus, showed a stronger output bias. Conversely, most input-biased regions were located in the isocortex, with the exception of the RHP in the HPF (Fig. 6b). Overall, connectivity tended to favor the ipsilateral hemisphere (Figs. S5, S6), consistent with our whole-brain level analysis. Notably, for contralateral connections, the VIS became output-biased, indicating a potential hemispheric asymmetry in visual processing (Fig. 6c, d). To enhance spatial understanding, we further segmented representative brain regions from the 3D whole-brain dataset (Fig. S4).

Soma labeling enabled detailed visualization of the brain-wide input-output architecture of the ACx. The connectivity patterns observed were both heterogeneous and topologically organized, with a clear predominance of ipsilateral projections (Videos S1, S2). Although many brain regions maintained bidirectional connections with the ACx, the strength of these connections varied considerably (Fig. 7a, b). By combining whole-brain registration and layer-specific analyses, we identified distinct spatial distribution patterns across multiple target areas (Fig. 4a, b). These results offer a more nuanced view of ACx connectivity and contribute to a better understanding of its role within large-scale brain networks.

**Fig. 7:**
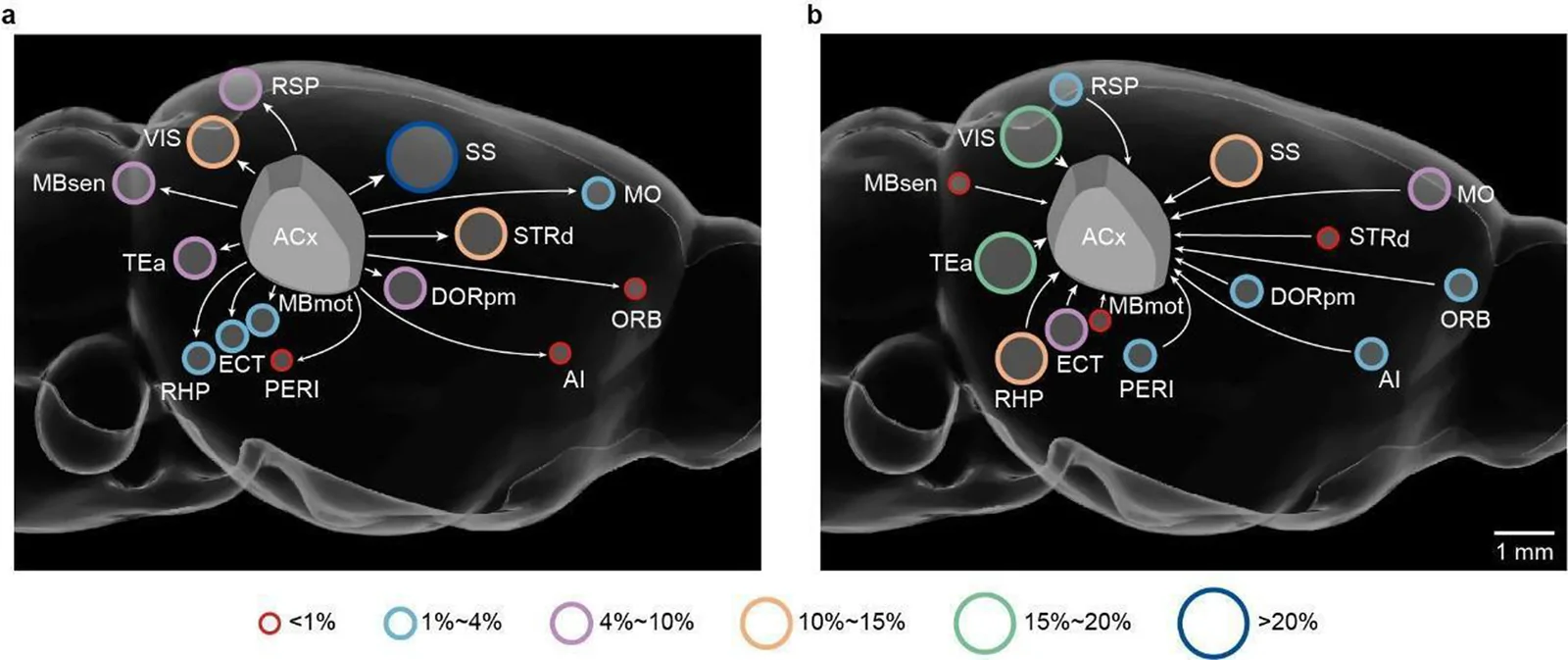
**Summary of brain-wide reciprocal connectivity.** (a) Summary of ACx outputs: Postsynaptic somata are predominantly restricted to the ipsilateral cortex. Dense projections are observed in the isocortex, STR, TH, and MB. (b) Schematic diagram illustrating the major inputs to ACx. Major input regions include the isocortex, HIP, and TH. Brain region demarcations and annotations were derived from the Allen Reference Atlas. Consistent color scales are applied across both panels to represent percentage values: < 1% (red), 1%–4% (light blue), 4%–10% (purple), 10%–15% (yellow), 15%–20% (green), and > 20% (dark blue).

While our soma-labeling strategy accurately maps mesoscale connectivity patterns, it does not resolve the anatomical or functional strength of connections (e.g., terminal bouton size or synaptic efficacy). For corticothalamic projections, where terminal size correlates with functional roles, complementary methods (e.g., electron microscopy or electrophysiology) would be needed to evaluate synaptic weights. Importantly, numerical connectivity does not necessarily predict functional impact, as sparse projections may still drive robust postsynaptic responses [38].

## 4 Conclusion

The auditory cortex is the central hub for sound processing, integrating bottom-up signals from the auditory periphery with top-down signals from other sensory and non-sensory brain areas [39,40].

In this study, we utilized an independently designed tracing system to label the outputs and inputs, revealing distinctions from previous findings. Firstly, we observed unique laminar characteristics; for example, in the SSp, layer 5 exhibited the densest projections, in contrast to previous reports indicating layers 1–3. Additionally, we identified outputs within the corpus callosum, the largest white matter structure in the brain, which connects nearly all the cerebral lobes [41]. Previous studies have highlighted its role in independent processing and information sharing, facilitating interhemispheric communication and reciprocal interactions between the visual cortex and callosal fibers [42,43]. The integrity of the corpus callosum is crucial for cognitive and motor functions, as well as restorative sleep [44,45].

By focusing on downstream somata rather than axonal terminals, our soma-labeling approach enabled a more precise depiction of brain-wide output distributions. This strategy helped to address the common limitation of difficulty distinguishing axonal terminals from passing fibers, which is typical in traditional tracing methods. Targeting somata also improved the efficiency and accuracy of layer-specific analyses. Overall, this work introduces a new perspective on whole-brain connectivity mapping, revealing previously unrecognized volumetric and laminar features. These insights advance our understanding of the ACx’s brain-wide network organization.

While our integrated viral tracing strategy provides a detailed brain-wide map of ACx connectivity, certain limitations inherent to viral labeling must be considered. First, although AAV serotypes (AAV1 and AAV2-retro) and neuron-specific promoters (hSyn) were employed to restrict expression to auditory cortical neurons, off-target labeling in nearby regions or non-neuronal cells remains a possibility. We validated injection sites and excluded samples with significant leakage (Fig. 2a, b), yet minor diffusion or ectopic expression in passing axons cannot fully be ruled out, introducing potential false positives. Additionally, while AAV1 is optimized for monosynaptic tracing, rare polysynaptic spread over prolonged expression windows may occur, potentially overestimating direct projections. Similarly, retrograde AAVs may capture axons of passage rather than exclusively synaptic inputs. To minimize these effects, we selected samples with tightly localized starter neurons and used nuclear-localized fluorescent proteins to improve somatic specificity over axonal background. Future studies combining intersectional genetics (e.g., Cre-dependent viruses in cell-type-specific lines) will help resolve these concerns. To maintain consistency with prior foundational studies [31], we used male mice in this initial work to establish baseline connectivity. However, we agree that sex differences may influence ACx connectivity. Future studies will include both sexes to assess potential sex-dependent variations.

In this study, we used the retrograde AAV variant (AAVRetro-EGFP) for input mapping, which has been demonstrated to perform comparably to classical cholera toxin subunit B (CTB) tracers in labeling projection neurons [12]. Beyond similar efficiency, AAVRetro-EGFP provides additional advantages, including its nuclear-localized signal confinement, which reduces the axonal background, and its viral nature enables prolonged expression for weeks to months, whereas CTB often fades within 1–2 weeks. These attributes make rAAV-retro a robust alternative for studying extended neural networks with high spatiotemporal precision.

The viral labeling strategy developed here, combining nuclear-localized elements with both retrograde and anterograde transsynaptic tracing, offers improved resolution for structural connectivity mapping and presents new opportunities for exploring functional neural circuits.

## Declaration of competing interest

The authors declare that they have no conflicts of interest in this work.

## Appendix Supplementary materials

Image, image 1 dummy alt text

Image, image 2 dummy alt text

Image, image 3 dummy alt text

Image, image 4 dummy alt text

Image, image 5 dummy alt text

Image, image 6 dummy alt text

Image, application 7

Image, application 8

Image, application 9

Image, application 10

Image, video 11 dummy alt text

Supplementary materials Image, video 12 dummy alt text
