# Supplementary material for: Brain-wide atlas of outputs and inputs of mouse auditory cortex revealed by modified viral labeling strategies
Source: Fundam Res. 2025 Sep 8;6(5):3047–55. doi: 10.1016/j.fmre.2025.08.014 (PMC13644523; doi:10.1016/j.fmre.2025.08.014)
Supplement: Supplementary file 1 [file mmc10.docx]

| Table S3 Number of antrogradely labeled neurons following Retro-AAV-mediated transsynatic tracing of inputs of auditory cortical neurons | | | | | | | | | | | | | | | |
| --- | --- | --- | --- | --- | --- | --- | --- | --- | --- | --- | --- | --- | --- | --- | --- |
|  |  | **Animal 1** | |  | **Animal 2** | |  | **Animal 3** | |  | **Animal 4** | |  | **Animal 5** | |
|  |  | **Ipsi** | **Contra** |  | **Ipsi** | **Contra** |  | **Ipsi** | **Contra** |  | **Ipsi** | **Contra** |  | **Ipsi** | **Contra** |
| Isocortex | ACA | 1909 | 578 |  | 2161 | 516 |  | 2061 | 655 |  | 1893 | 636 |  | 2004 | 310 |
|  | AI | 3002 | 272 |  | 2878 | 575 |  | 2554 | 555 |  | 2206 | 545 |  | 2751 | 294 |
|  | ECT | 12030 | 2092 |  | 13777 | 3247 |  | 12825 | 3213 |  | 12274 | 2224 |  | 12698 | 2709 |
|  | FRP | 0 | 0 |  | 3 | 2 |  | 0 | 0 |  | 20 | 10 |  | 30 | 15 |
|  | GU | 113 | 26 |  | 97 | 5 |  | 136 | 6 |  | 198 | 30 |  | 149 | 12 |
|  | ILA | 48 | 1 |  | 64 | 6 |  | 43 | 5 |  | 40 | 7 |  | 62 | 8 |
|  | MO | 8371 | 1928 |  | 9703 | 2056 |  | 10447 | 1553 |  | 10683 | 2283 |  | 9025 | 1727 |
|  | ORB | 5282 | 802 |  | 5507 | 497 |  | 5129 | 733 |  | 5666 | 471 |  | 5423 | 436 |
|  | PERI | 4712 | 790 |  | 4899 | 1067 |  | 4854 | 892 |  | 4752 | 733 |  | 4981 | 812 |
|  | PL | 46 | 10 |  | 62 | 13 |  | 61 | 10 |  | 278 | 159 |  | 679 | 165 |
|  | RSP | 6280 | 525 |  | 6746 | 462 |  | 6401 | 522 |  | 6859 | 570 |  | 6495 | 560 |
|  | SS | 41875 | 3157 |  | 39236 | 3985 |  | 40462 | 3402 |  | 41418 | 3512 |  | 42018 | 3497 |
|  | TEa | 47579 | 7087 |  | 47098 | 7285 |  | 46284 | 8012 |  | 48039 | 7131 |  | 47293 | 7098 |
|  | VIS | 37928 | 2651 |  | 39671 | 2882 |  | 40654 | 2551 |  | 39019 | 3222 |  | 39489 | 2916 |
|  | VISC | 2090 | 89 |  | 2123 | 68 |  | 2049 | 83 |  | 1902 | 77 |  | 1824 | 75 |
| Olfactory Areas | AOB | 0 | 0 |  | 0 | 0 |  | 13 | 0 |  | 0 | 0 |  | 0 | 0 |
|  | AON | 18 | 2 |  | 8 | 0 |  | 9 | 4 |  | 13 | 1 |  | 15 | 5 |
|  | MOB | 320 | 4 |  | 275 | 6 |  | 293 | 2 |  | 300 | 1 |  | 263 | 2 |
|  | TT | 102 | 4 |  | 88 | 6 |  | 104 | 10 |  | 92 | 7 |  | 95 | 7 |
|  | COA | 899 | 108 |  | 1013 | 95 |  | 912 | 88 |  | 994 | 47 |  | 946 | 82 |
|  | DP | 8 | 1 |  | 9 | 1 |  | 10 | 1 |  | 9 | 0 |  | 8 | 0 |
|  | NLOT | 8 | 2 |  | 8 | 1 |  | 13 | 0 |  | 13 | 0 |  | 8 | 0 |
|  | PAA | 140 | 21 |  | 166 | 17 |  | 157 | 27 |  | 148 | 22 |  | 161 | 26 |
|  | PIR | 1494 | 165 |  | 1703 | 627 |  | 1990 | 464 |  | 1107 | 528 |  | 1507 | 158 |
|  | TR | 1501 | 57 |  | 685 | 12 |  | 509 | 27 |  | 496 | 20 |  | 554 | 11 |
| HPF | HIP | 1987 | 13 |  | 1983 | 17 |  | 1890 | 18 |  | 908 | 13 |  | 1891 | 14 |
|  | RHP | 33377 | 3865 |  | 32974 | 3569 |  | 34848 | 3504 |  | 33242 | 3901 |  | 37048 | 3916 |
|  | CTXsp | 5287 | 610 |  | 5542 | 682 |  | 5306 | 674 |  | 5540 | 628 |  | 5384 | 618 |
| Striatum | STRd | 426 | 56 |  | 412 | 66 |  | 422 | 64 |  | 449 | 78 |  | 447 | 72 |
|  | STRv | 27 | 10 |  | 30 | 1 |  | 30 | 2 |  | 25 | 1 |  | 27 | 2 |
|  | LSX | 5 | 0 |  | 7 | 0 |  | 5 | 0 |  | 7 | 0 |  | 4 | 0 |
|  | sAMY | 56 | 4 |  | 54 | 7 |  | 60 | 10 |  | 56 | 8 |  | 51 | 2 |
| Pallidum | PALd | 28 | 1 |  | 26 | 3 |  | 33 | 4 |  | 31 | 4 |  | 30 | 5 |
|  | PALv | 26 | 4 |  | 31 | 6 |  | 25 | 0 |  | 28 | 0 |  | 31 | 4 |
|  | PALm | 10 | 1 |  | 10 | 3 |  | 11 | 4 |  | 8 | 0 |  | 5 | 0 |
|  | PALc | 3 | 0 |  | 6 | 0 |  | 0 | 0 |  | 2 | 0 |  | 5 | 0 |
| Thalamus | DORsm | 12195 | 34 |  | 10497 | 32 |  | 10964 | 42 |  | 10811 | 47 |  | 10206 | 37 |
|  | DORpm | 5721 | 133 |  | 5739 | 78 |  | 5917 | 114 |  | 6160 | 140 |  | 6088 | 152 |
| Hypothalamus | PVZ | 10 | 0 |  | 10 | 2 |  | 16 | 2 |  | 16 | 1 |  | 16 | 0 |
|  | PVR | 10 | 1 |  | 19 | 6 |  | 11 | 0 |  | 14 | 7 |  | 12 | 6 |
|  | MEZ | 218 | 25 |  | 210 | 26 |  | 206 | 21 |  | 230 | 26 |  | 229 | 23 |
|  | LZ | 135 | 16 |  | 129 | 14 |  | 123 | 11 |  | 140 | 19 |  | 131 | 20 |
| Midbrain | Mbsen | 381 | 84 |  | 375 | 33 |  | 364 | 79 |  | 377 | 50 |  | 387 | 53 |
|  | Mbmot | 830 | 93 |  | 857 | 87 |  | 867 | 97 |  | 849 | 73 |  | 813 | 91 |
|  | Mbsta | 22 | 3 |  | 27 | 6 |  | 26 | 10 |  | 22 | 7 |  | 26 | 8 |
| Pons | P-sen | 64 | 10 |  | 70 | 11 |  | 68 | 10 |  | 73 | 12 |  | 70 | 9 |
|  | P-mot | 2 | 0 |  | 3 | 0 |  | 4 | 0 |  | 2 | 0 |  | 3 | 0 |
|  | P-sat | 50 | 14 |  | 37 | 10 |  | 46 | 11 |  | 45 | 12 |  | 49 | 7 |
| Medulla | MY-sen | 0 | 0 |  | 4 | 2 |  | 2 | 0 |  | 4 | 2 |  | 6 | 2 |
|  | MY-mot | 5 | 1 |  | 2 | 0 |  | 1 | 0 |  | 1 | 0 |  | 7 | 1 |
|  | MY-sat | 3 | 1 |  | 2 | 1 |  | 4 | 1 |  | 0 | 0 |  | 4 | 2 |
|  | CB | 248 | 26 |  | 233 | 17 |  | 224 | 21 |  | 240 | 26 |  | 204 | 26 |
|  | fiber tracts | 10383 | 105 |  | 8352 | 106 |  | 3972 | 107 |  | 3669 | 136 |  | 10637 | 318 |
