# Supplementary material for: Brain-wide atlas of outputs and inputs of mouse auditory cortex revealed by modified viral labeling strategies
Source: Fundam Res. 2025 Sep 8;6(5):3047–55. doi: 10.1016/j.fmre.2025.08.014 (PMC13644523; doi:10.1016/j.fmre.2025.08.014)
Supplement: Supplementary file 2 [file mmc9.docx]

| Table S2 Number of antrogradely labeled neurons following AAV-mediated transsynatic tracing of outputs from auditory cortical neurons | | | | | | | | | | | | | | | |
| --- | --- | --- | --- | --- | --- | --- | --- | --- | --- | --- | --- | --- | --- | --- | --- |
|  |  | **Animal 1** | |  | **Animal 2** | |  | **Animal 3** | |  | **Animal 4** | |  | **Animal 5** | |
|  |  | **Ipsi** | **Contra** |  | **Ipsi** | **Contra** |  | **Ipsi** | **Contra** |  | **Ipsi** | **Contra** |  | **Ipsi** | **Contra** |
| Isocortex | ACA | 4341 | 377 |  | 4315 | 363 |  | 4229 | 409 |  | 4180 | 354 |  | 4065 | 361 |
|  | AI | 1436 | 360 |  | 1441 | 354 |  | 1425 | 306 |  | 1455 | 398 |  | 1373 | 367 |
|  | ECT | 17761 | 2835 |  | 17707 | 2633 |  | 18437 | 2923 |  | 19918 | 2666 |  | 18797 | 2850 |
|  | FRP | 102 | 10 |  | 112 | 15 |  | 109 | 19 |  | 114 | 13 |  | 108 | 15 |
|  | GU | 148 | 18 |  | 145 | 17 |  | 138 | 17 |  | 161 | 16 |  | 147 | 11 |
|  | ILA | 140 | 6 |  | 144 | 5 |  | 131 | 8 |  | 135 | 10 |  | 130 | 9 |
|  | MO | 16363 | 1036 |  | 15033 | 1232 |  | 16069 | 1394 |  | 15216 | 1224 |  | 15819 | 1281 |
|  | ORB | 4119 | 646 |  | 4804 | 610 |  | 4456 | 595 |  | 4295 | 697 |  | 4209 | 634 |
|  | PERI | 2080 | 707 |  | 1857 | 783 |  | 2043 | 798 |  | 1969 | 712 |  | 2055 | 794 |
|  | PL | 674 | 150 |  | 721 | 120 |  | 689 | 139 |  | 670 | 91 |  | 674 | 106 |
|  | RSP | 24612 | 2690 |  | 24102 | 2603 |  | 22283 | 2525 |  | 23114 | 2496 |  | 23387 | 2704 |
|  | SS | 119322 | 12664 |  | 122435 | 12464 |  | 124979 | 2733 |  | 128106 | 12680 |  | 125652 | 11401 |
|  | TEa | 39464 | 9775 |  | 41938 | 10110 |  | 40302 | 10641 |  | 42249 | 11131 |  | 40543 | 10377 |
|  | VIS | 53221 | 6268 |  | 49853 | 6003 |  | 50764 | 6381 |  | 55545 | 6877 |  | 52367 | 6716 |
|  | VISC | 3738 | 516 |  | 3809 | 561 |  | 4154 | 522 |  | 4179 | 601 |  | 3907 | 586 |
| Olfactory Areas | AOB | 0 | 0 |  | 8 | 0 |  | 0 | 0 |  | 0 | 0 |  | 0 | 0 |
|  | AON | 69 | 20 |  | 71 | 12 |  | 77 | 22 |  | 69 | 17 |  | 76 | 13 |
|  | MOB | 300 | 56 |  | 374 | 65 |  | 303 | 62 |  | 301 | 60 |  | 391 | 85 |
|  | TT | 101 | 32 |  | 107 | 26 |  | 131 | 36 |  | 121 | 30 |  | 115 | 20 |
|  | COA | 86 | 5 |  | 79 | 8 |  | 101 | 8 |  | 90 | 4 |  | 85 | 4 |
|  | DP | 154 | 10 |  | 132 | 12 |  | 124 | 11 |  | 138 | 9 |  | 126 | 9 |
|  | NLOT | 4 | 0 |  | 5 | 0 |  | 4 | 0 |  | 6 | 0 |  | 3 | 0 |
|  | PAA | 43 | 17 |  | 45 | 13 |  | 61 | 12 |  | 50 | 13 |  | 53 | 13 |
|  | PIR | 334 | 155 |  | 352 | 171 |  | 363 | 164 |  | 351 | 167 |  | 370 | 164 |
|  | TR | 109 | 2 |  | 113 | 4 |  | 108 | 9 |  | 116 | 7 |  | 97 | 1 |
| HPF | HIP | 731 | 261 |  | 707 | 228 |  | 765 | 262 |  | 748 | 282 |  | 741 | 243 |
|  | RHP | 9735 | 1843 |  | 10790 | 1799 |  | 11422 | 1753 |  | 11853 | 1807 |  | 8428 | 1822 |
|  | CTXsp | 7795 | 610 |  | 7607 | 679 |  | 7693 | 600 |  | 8160 | 614 |  | 7975 | 548 |
| Striatum | STRd | 79802 | 6198 |  | 75392 | 6476 |  | 76662 | 6729 |  | 76144 | 6419 |  | 79447 | 6597 |
|  | STRv | 1704 | 362 |  | 1747 | 309 |  | 1657 | 340 |  | 1750 | 225 |  | 1734 | 236 |
|  | LSX | 91 | 18 |  | 86 | 22 |  | 84 | 19 |  | 91 | 20 |  | 86 | 19 |
|  | sAMY | 3271 | 96 |  | 3507 | 104 |  | 3550 | 82 |  | 3679 | 113 |  | 3784 | 114 |
| Pallidum | PALd | 1404 | 63 |  | 1355 | 52 |  | 1277 | 77 |  | 1416 | 47 |  | 1369 | 48 |
|  | PALv | 588 | 38 |  | 511 | 29 |  | 519 | 26 |  | 471 | 35 |  | 502 | 25 |
|  | PALm | 86 | 27 |  | 94 | 25 |  | 111 | 24 |  | 97 | 29 |  | 79 | 30 |
|  | PALc | 69 | 7 |  | 81 | 6 |  | 66 | 10 |  | 70 | 8 |  | 55 | 7 |
| Thalamus | DORsm | 24544 | 408 |  | 24527 | 376 |  | 25103 | 284 |  | 24138 | 190 |  | 26098 | 177 |
|  | DORpm | 28284 | 237 |  | 27974 | 199 |  | 28303 | 224 |  | 29297 | 265 |  | 31632 | 232 |
| Hypothalamus | PVZ | 162 | 28 |  | 196 | 30 |  | 177 | 26 |  | 186 | 25 |  | 195 | 49 |
|  | PVR | 526 | 12 |  | 555 | 29 |  | 498 | 48 |  | 537 | 20 |  | 556 | 20 |
|  | MEZ | 1388 | 68 |  | 1447 | 51 |  | 1435 | 78 |  | 1422 | 115 |  | 1444 | 93 |
|  | LZ | 2353 | 60 |  | 2591 | 69 |  | 2481 | 58 |  | 2562 | 60 |  | 2576 | 55 |
| Midbrain | Mbsen | 21723 | 2697 |  | 20795 | 2423 |  | 20611 | 2822 |  | 25330 | 2515 |  | 23471 | 2673 |
|  | Mbmot | 17819 | 2393 |  | 18183 | 2172 |  | 16901 | 2081 |  | 16701 | 1924 |  | 16432 | 2293 |
|  | Mbsta | 540 | 58 |  | 560 | 99 |  | 439 | 82 |  | 508 | 108 |  | 568 | 57 |
| Pons | P-sen | 1482 | 275 |  | 1499 | 186 |  | 1539 | 371 |  | 1479 | 260 |  | 1478 | 288 |
|  | P-mot | 2168 | 386 |  | 2215 | 382 |  | 2019 | 376 |  | 2175 | 362 |  | 2069 | 339 |
|  | P-sat | 699 | 369 |  | 567 | 135 |  | 790 | 232 |  | 936 | 133 |  | 782 | 143 |
| Medulla | MY-sen | 186 | 75 |  | 227 | 71 |  | 172 | 51 |  | 180 | 66 |  | 185 | 74 |
|  | MY-mot | 310 | 45 |  | 432 | 38 |  | 532 | 53 |  | 456 | 50 |  | 424 | 40 |
|  | MY-sat | 22 | 23 |  | 31 | 13 |  | 23 | 8 |  | 55 | 23 |  | 33 | 13 |
|  | CB | 423 | 89 |  | 579 | 122 |  | 436 | 115 |  | 423 | 104 |  | 690 | 152 |
|  | fiber tracts | 18516 | 3953 |  | 17176 | 4195 |  | 15198 | 3615 |  | 17711 | 3352 |  | 18252 | 3488 |
