# Supplementary material for: Brain-wide atlas of outputs and inputs of mouse auditory cortex revealed by modified viral labeling strategies
Source: Fundam Res. 2025 Sep 8;6(5):3047–55. doi: 10.1016/j.fmre.2025.08.014 (PMC13644523; doi:10.1016/j.fmre.2025.08.014)
Supplement: Supplementary file 4 [file mmc8.doc]

**Table S1 Abbreviations of brain regions.**
